# Measurement of the Effects of School Psychological Services: A Scoping Review

**DOI:** 10.3389/fpsyg.2021.606228

**Published:** 2021-04-16

**Authors:** Bettina Müller, Alexa von Hagen, Natalie Vannini, Gerhard Büttner

**Affiliations:** Department of Educational Psychology, Competence Centre School Psychology Hesse, Institute for Psychology, Goethe University Frankfurt, Frankfurt am Main, Germany

**Keywords:** efficacy, evaluation, school psychology, scoping review, measurement

## Abstract

School psychologists are asked to systematically evaluate the effects of their work to ensure quality standards. Given the different types of methods applied to different users of school psychology measuring the effects of school psychological services is a complex task. Thus, the focus of our scoping review was to systematically investigate the state of past research on the measurement of the effects of school psychological services published between 1998 and 2018 in eight major school psychological journals. Of the 5,048 peer-reviewed articles published within this period, 623 were coded by two independent raters as explicitly refering to *school psychology* or *counseling* in the school context in their titles or abstracts. However, only 22 included definitions of effects of school psychological services or described outcomes used to evaluate school psychological services based on full text screening. These findings revealed that measurement of the effects of school psychological services has not been a focus of research despite its' relevance in guidelines of school psychological practice.

## Introduction

School psychology is an independent and applied field of psychology concerned with providing mental health services to students, their families, teachers, school principals and other school staff (Jimerson et al., 2007; Bundesverband Deutscher Psychologinnen und Psychologen, 2015; American Psychological Association, 2020). According to the APA “school psychologists are prepared to intervene at the individual and system level, and develop, implement and evaluate programs to promote positive learning environments for children and youth from diverse backgrounds, and to ensure equal access to effective educational and psychological services that promote health development” (American Psychological Association, 2020). They promote “healthy growth and development of children and youth in a range of contexts” that impact instruction, learning or school behavior by providing services such as “assessment and evaluation of individuals and instructional and organizational environments,” “prevention and intervention programs,” “crisis intervention,” “consultations with teachers, parents, administrators, and other health service providers,” “supervision of psychological services” and “professional development programs” (American Psychological Association, 2020).

While a large extent of the available scientific literature on the scope of school psychology is consistent with the above-mentioned definition of the APA and reflects common practice in the U.S., there are considerable differences among countries (see Jimerson et al., 2007 for an extensive overview of school psychology across 43 countries). In coherence, the International School Psychology Association (ISPA) states that “the term school psychology is used in a general form to refer to professionals prepared in psychology and education and who are recognized as specialists in the provision of psychological services to children and youth within the context of schools, families, and other settings that impact their growth and development. As such, the term also refers to and is meant to include educational psychologists (…)” (International School Psychology Association, 2021). For example, in England, Wales, and Hong Kong the term “educational psychologists” is used as an equivalent to the term school psychologists (Lam, 2007; Squires and Farrell, 2007). In this review we use the term “school psychology” in coherence with the above-mentioned definition provided by the International School Psychology Association (2021).

A particular characteristic of school psychology is its multifaceted nature. Practitioners in this discipline cater for the needs of different types of users (e.g., students, teachers, parents) by relying on diverse methods (e.g., counseling of individuals or groups of students or teachers, screening and diagnostics, training, consultation). Moreover, school psychologists address a broad range of psychological needs (e.g., academic achievement, mental health, and behavior) and support conducive learning environments and practices (Bundesverband Deutscher Psychologinnen und Psychologen, 2015; American Psychological Association, 2020). They provide student-level services (i.e., educational and mental health interventions and services offered for small groups or individuals) as well as system-level services (e.g., implementation and evaluation of programs for teachers). *Student-level services* are, however, not limited to direct interventions with students. In order to support students effectively, it is often necessary to work with adults that play a significant role in students' lives. School psychologists, therefore, often rely on indirect services with parents and teachers to promote well-being in students. These mediated actions that underlie indirect service delivery models of school psychology are often referred to as the “*paradox of school psychology*” following Gutkin and Conoley (1990). Again, there are considerable differences among and within countries with respect to the extent that school psychologists engage in these direct and indirect services. For instance, in some regions of the U.S. school psychologists are mostly responsible for providing direct services like psychoeducational assessments, while a related professional group of so-called “*school counselors*” engage in indirect services such as consultation. In contrast, in a study by Bahr et al. (2017) school psychologists from three Midwestern U.S. states reported that “problem-solving consultation was the activity on which they spent the greatest amount of their time” (p. 581). Recent developments have extended the role of school psychologists to also provide *system-level services* that aim to support the organizational development of schools. In this context, a lot of emphasis is being placed on preventive work and interventions with parents, educators, and other professionals that intend to create supportive learning and social environments for students (Burns, 2011; Skalski et al., 2015).

Professional associations in different countries have attempted to summarize the above-mentioned multi-faceted nature of school psychology in practical frameworks and/or models. For instance, the *Model for Services by School Psychologists* by the National Association of School Psychology in the U.S. (Skalski et al., 2015) distinguishes between student-level services (i.e., interventions and instructional support to develop academic skills and interventions and mental health services to develop social and life skills) and systems-level services (i.e., school-wide practices to promote learning, prevention, responsive services and family-school collaboration services). Similarly, the Department of School Psychology of the Professional Association of German Psychologists (Bundesverband Deutscher Psychologinnen und Psychologen, 2015) states that school psychologists provide support for students through individual counseling in cases of learning, developmental, and behavioral problems of students (e.g., fostering gifted students, identifying special needs in inclusive schools, etc.), as well as for schools through system-level consultation (e.g., development toward inclusive schools, violence prevention, etc.).

There have also been several theoretical proposals to conceptualize the manifold field of school psychology. For example, Nastasi (2000) suggested that this subdiscipline should be understood as a comprehensive health care service that ranges from prevention to treatment. Also, Sheridan and Gutkin (2000) advocated for an ecological framework of service delivery that takes into account multiple eco-systemic levels. Moreover, Strein et al. (2003) acknowledged that thinking of school psychology in terms of direct and indirect services has advanced the field notably. They suggest broadening the framework even further to adopt a public health perspective that evaluates both individual- and system-level outcomes. Although there are, undoubtably, many differences between the way school psychology is understood and practiced in different countries, there seems to be consensus that the broad distinctions between individual vs. system-level and direct vs. indirect services represent an essential part of the scope of school psychological services (cf. Jimerson et al., 2007).

Just like other health professionals, school psychologists are expected to rely on evidence-based practices and evaluate the effects of their interventions to ensure quality standards (Hoagwood and Johnson, 2003; Kratochwill and Shernoff, 2004; White and Kratochwill, 2005; Forman et al., 2013; Morrison, 2013). For instance, some of the examples of professional practice included in the domain of Research and Program Evaluation of the *Model for Services by School Psychologists* by the NASP (Skalski et al., 2015) in the U.S. are “using research findings as the foundation for effective service delivery” and “using techniques of data collection to evaluate services at the individual, group, and systems levels” (Skalski et al., 2015, p. I-5). Similarly, the professional profile of the Department of School Psychology of the Professional Association of German Psychologists (Bundesverband Deutscher Psychologinnen und Psychologen, 2015) mentions that school psychologists engage in regular measures of documentation, evaluation and quality insurance of their work in collaboration with their superiors (Bundesverband Deutscher Psychologinnen und Psychologen, 2015, p. 6).

Measuring the effects of service delivery is, however, a very complex task that needs to encompass the multiple dimensions involved in school psychological practice, if we take into consideration the multifaceted nature of school psychology described above. This makes it difficult to define the effects of school psychological services and to derive recommendations on how to operationalize and measure them in practice. Practical guidelines and/or models, such as the ones mentioned above, rarely provide any specifications about the designs, instruments, or outcome variables that should be used to implement service evaluations. Results of a survey on contemporary practices in U.S. school psychological services showed that 40% reported using teacher or student reports (verbal and written), observational data, and single-subject design procedures to evaluate their services (Bramlett et al., 2002). In contrast, the results of the International School Psychology Survey indicate that this is not the international standard. School psychologists in many countries complain about the lack of research and evaluation of school psychological services in their country (Jimerson et al., 2004) or even express the need for more studies on service evaluation (Jimerson et al., 2006). Although the survey did not collect information about (self-)evaluation practices, results suggest huge variability in evaluation and thereby the understanding of effects of school psychological services.

Furthermore, attempts to define the term “effects of school psychological services” vary considerably and imply different ways of operationalizing measurement of effects. Some approaches define effects as significant changes in outcome variables that are thought to be a consequence of service delivery. Other approaches define effects as the impact of interventions as perceived by school psychologists themselves and/or clients (i.e., consumer satisfaction) or follow an economic point of view describing effects in relation to costs. These diverse perspectives seem to be used in parallel or even as synonyms, although they refer to different aspects of evaluation of school psychological services. For instance, Phillips (1998) suggested that the methods used by practicing school psychologist need to be investigated through controlled experimental evaluation studies conducted by researchers to determine changes in students, teachers, and/or other users of services. In this perspective an effect refers to the extent to which services can achieve the expected outcome. According to Andrews (1999) this should be labeled as efficacy and measured in experimental settings, ideally randomized controlled trials. In contrast to standardized designs commonly used in empirical research, school psychological practices are often characterized by the simultaneous application of heterogeneous methods to a broad range of psychological needs with varying frequencies. Thus, as a next step, effective methods that showed significant results in experimental designs need to be applied by school psychologists in practice to confirm their effectiveness in real-world settings where ideal conditions cannot be assured (Andrews, 1999; White and Kratochwill, 2005; Forman et al., 2013).

From a different perspective, some authors define the effects of school psychological services as the perceived impact of services on students, teachers, and/or other users from the perspective of school psychologists (e.g., Manz et al., 2009) or from the users' perspectives (e.g., Sandoval and Lambert, 1977; Anthun, 2000; Farrell et al., 2005). Again, another group of studies argue that evaluation results are necessary to justify cost-effectiveness of school psychological services. For example, Phillips (1998) stated that the “value of psychological service is measured by its efficiency, i.e., its desirable outcomes in relation to its costs” (p. 269; cf. Andrews, 1999). For this purpose, effects are often operationalized via frequency counts, like the number of tests used or number of children screened for special education per month (Sandoval and Lambert, 1977), the number of students participating in services offered by school psychologists (Braden et al., 2001), or the time dedicated to certain activities.

Taken together, what exactly is meant when school psychologists are asked to systematically evaluate the effects of their work and in this way ensure quality standards, seems to depend on the (often implicit) definitions and operationalizations of the measurement of effects each guideline or model adheres to. A possible reason for this variability might be related to the broad scope of the field of school psychology. As described in the *paradox of school psychology* (Gutkin and Conoley, 1990) it is mostly necessary to work with adults playing significant roles in the students' life to support students effectively. Thus, the impact of school psychological services on students is often indirect, mediated by actions of adults like teachers or parents who interact directly with school psychologists (Gutkin and Curtis, 2008). This poses methodological challenges for the measurement of effects of school psychological services, since both the development of the students as well as the changes in adults can be used as outcomes to define and operationalize effects in the three aforementioned perspectives.

One way of shedding light on this matter is to rely on evidence synthesis methodologies to reach a better understanding of how effects of school psychological services have been conceptualized to date, as Burns et al. (2013) propose. These authors conducted a mega-analysis summarizing past research from 47 meta-analyses to inform practice and policy on the effectiveness of school psychological services. While their findings reveal moderate and large effects for various types of school psychological services, several questions remain unanswered with respect to the measurement of the above-mentioned effects. Burns et al. (2013) charted the available evidence with great detail focusing on the type of intervention (e.g., domain of practice: academic, social and health wellness, etc.; tier of intervention: universal, targeted, intensive, etc.) and target population (e.g., preschool, elementary, middle or high school students). However, their focus did not lie on measurement of effects itself, such as distinguishing between effects measured from the perspective of school psychologists, service users or objective assessment measures. This is important, because the efficacy of an intervention might depend on the measurement used to capture results (e.g., consumer satisfaction survey vs. standardized test). Furthermore, the evidence summarized by Burns et al. (2013) was not limited to research explicitly conducted in the field of school psychology, but rather in the broader context of educational psychology. While there are undoubtably several relevant studies that nurture the field of school psychology without explicitly mentioning it, it is also important to highlight research that explicitly focuses on school psychology to strengthen the adoption of evidence-based practices in this field.

Building on this work, in the present review we aimed to address these gaps and contribute to a better understanding of the measurement of effects of school psychological services by systematically investigating the state of past research on this topic. We decided to adopt a scoping review approach, a type of knowledge synthesis to summarize research findings, identify the main concepts, definitions, and studies on a topic as well as to determine research gaps to recommend or plan future research (Peters et al., 2015; Tricco et al., 2018). Compared to systematic reviews (e.g., Liberati et al., 2009) scoping reviews mostly address a broader research question and summarize different types of evidence based on heterogeneous methods and designs. There is no need to examine the risk of bias of the included evidence to characterize the extent and content of research to a topic (Peters et al., 2015; Tricco et al., 2018). Thus, scoping reviews can be used to generate specific research questions and hypotheses for future systematic reviews (Tricco et al., 2016). Our review was guided by four research questions: (1) What percentage of articles in these journals focus on the measurement of effects of school psychological services? (2) What type of articles (i.e., empirical, theoretical/conceptual, meta-analysis) have been published on this topic and what is their content? (3) How did the authors define effects of school psychological services in past research? and (4) Which instruments are used to operationalize measurement of effects?

## Methods and Procedure

We followed the methodological guidelines suggested by the PRISMA guidelines for scoping reviews (PRISMA-ScR, Tricco et al., 2018) and provide details in the following sections.

### Eligibility Criteria

We followed a similar procedure as Villarreal et al. (2017) and limited our search to eight major peer-reviewed school psychology journals: *Canadian Journal of School Psychology, International Journal of School and Educational Psychology, Journal of Applied School Psychology, Journal of School Psychology, Psychology in the School, School Psychology International, School Psychology Quarterly*, and *School Psychology Review*. Furthermore, following common practices in systematic review methodology, we focused on articles published between 1998 (the year after the National Association of School Psychologists of the U.S. published the Blueprint for Training to guide training and practice, Yesseldyke et al., 1997) and September 2018. In this way, we aimed to capture past research published in the last 20 years. We only focused on reports written in English, as the review team is not proficient enough in other languages (e.g., French reports in the *Canadian Journal of School Psychology*) to assess eligibility for this review.

Empirical research studies (i.e., articles reporting new data from quantitative or qualitative studies using either a sample of school psychologists or a sample of teachers, principals, students or other users of school psychological services), theoretical/conceptual contributions (i.e., articles on school psychological services based on the literature), or meta-analyses/systematic review (i.e., articles systematically analyzing results from multiple studies within the field of school psychology) were included. Editorials, commentaries, book and test reviews were excluded. Furthermore, to meet eligibility criteria for this review, articles had to explicitly refer to “school psychology” or “counseling” in the school context in their title and/or abstract and needed to address effects of school psychological services by naming at least one of the following keywords in the abstract: “evaluation/evaluate,” “effect/effects,” “effectivity,” “efficacy” or “effectiveness.” Abstracts on school psychology with text that was limited to the discussion (e.g., “consequences for school psychology will be discussed”) were excluded. To address our third and fourth research question, at full-text screening stage only articles that provided definitions and/or operationalizations of the effects of school psychological services were included.

To identify the range of evidence published on the effects of school psychological services we included studies delivered with school psychologists as well as articles related to all kinds of direct and indirect services. No selection limitations were imposed about method, design, participants, or outcomes of the studies.

### Information Sources, Search, and Selection Process

The first author and a research assistant hand screened the journals' homepages from June to September 2018^1^. The selection process consisted of three steps. First, screening of titles and abstracts of all published articles was completed through independent double ratings by the first author and a research assistant to identify papers explicitly related to school psychology (search strategy: “school psychology” OR “counseling” in the school context). Articles were coded as included or excluded in an excel sheet. Moreover, the included articles were coded as empirical, theoretical/conceptual, or meta-analysis/systematic review. Inter-rater reliability values based on Cohen's kappa were estimated with the software package *psych* for R (Revelle, 2018). Second, the abstracts of the selected articles, even those with inclusion ratings from only one rater, were screened once again by the first author to identify articles on effects related to school psychological services (search strategy: evaluate^*^ OR effect^*^ OR effect^*^ OR efficacy). Third, full texts of all included articles were screened by the first author.

If access to full texts was restricted through the journals' homepages, we used electronic databases (e.g., PsychArticles), search engines (e.g., Google), interlibrary loan services (e.g., Subito documents library), or contacted the authors via ResearchGate to gain full access.

### Data Charting Process, Data Items, and Synthesis of Results

The first author charted data from the included articles in an excel sheet consisting of the following data items: type of article (i.e., empirical, theoretical/conceptual, or meta-analytical), authors, year of publication, content (i.e., summary of the study objective, participants and country of data collection or context referred to), definition of effects of school psychological services (i.e., explanation of outcomes used or recommended to measure effects of school psychological services), and operationalization of effects of school psychological services (i.e., instruments used or recommended to measure outcomes). Table 1 provides further details on each of these data items.

**Table 1:** Definitions of data items.

**Data item**	**Definition**
**Type of article**	
Empirical	The article reports on empirical data collected specifically in this study or on a secondary analysis of an existing dataset.
Theoretical/conceptual	The article does not report on an empirical research study, but rather proposed a theoretical model or framework to guide future research or practice.
Meta-analytical	The article systematically summarizes the available evidence across a set of empirical research studies.
Authors	All author names as mentioned in the article.
Year of publication	Year the study was published in the target journal as mentioned in the article.
**Content**	
Summary of the study objective	Aims or objectives of the study or article as expressed by the authors.
Participants	Type of providers or users of school psychological services targeted by the article (e.g., students, parents, teachers, etc.).
Country of data collection	Country the data was collected in or restriction of eligibility criteria to studies from certain countries for meta-analysis.
**Context referred to**	
Definition of effects of school psychological services	Outcomes used or recommended to measure effects of school psychological services
Operationalization of effects of school psychological services	Instruments used or recommended to measure outcomes of school psychological services

Based on the data charting form, we summarized our findings in a narrative manner and identified research clusters and gaps addressing our research questions. Specifically, for the category “effects of school psychological services,” we proposed a first categorization into thematic clusters grouping different types of definitions, based on the information we extracted from full texts during data charting. This categorization was then double-checked by revising the full texts to make sure that each of the included articles was accurately allocated to one of the proposed thematic clusters.

## Results

### Search and Selection Process

**Figure 1:**
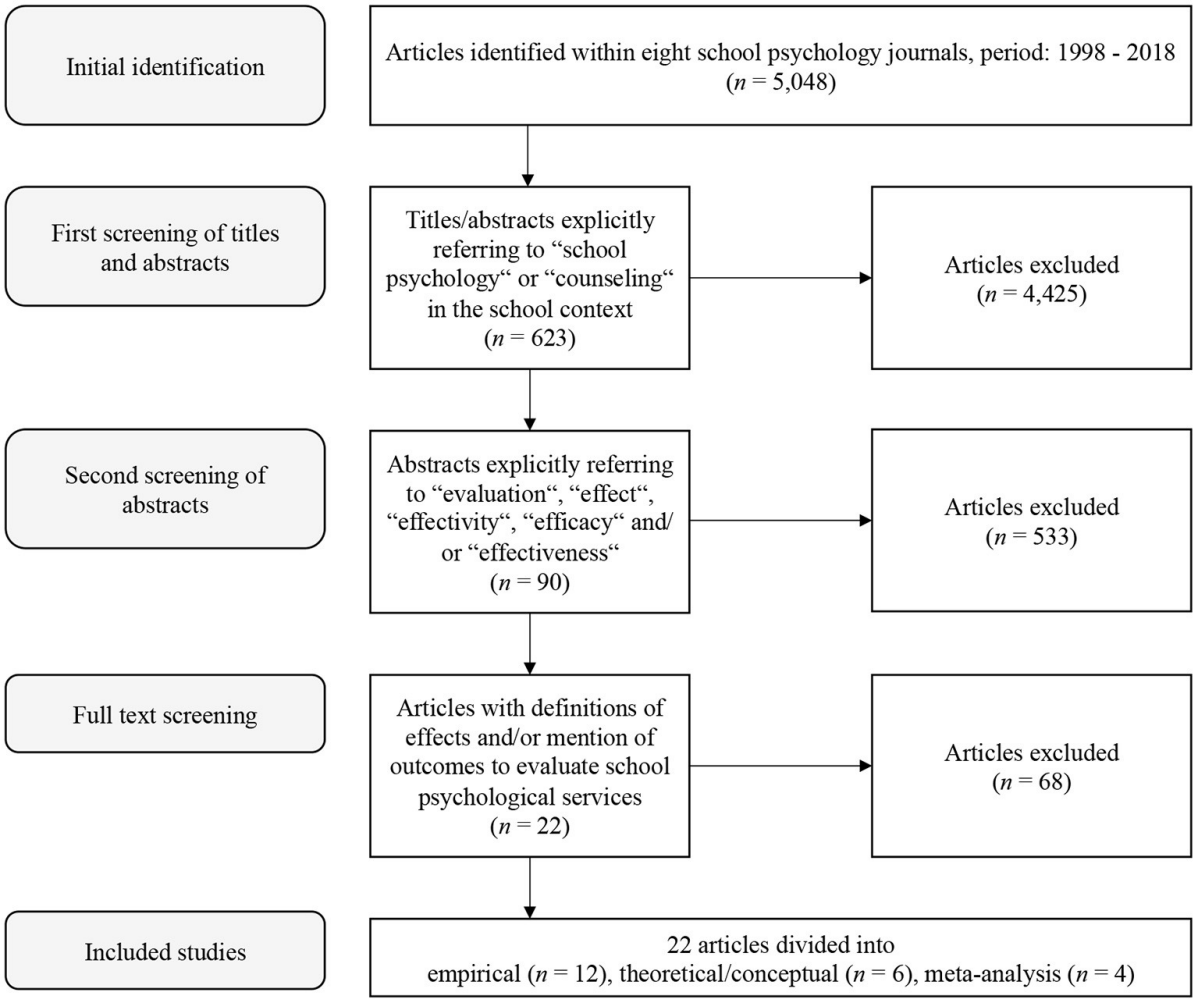
Flowchart of the scoping review procedure.

Figure 1 reveals the results of the search and selection process. Departing from 5,048 references that we initially identified, 4,425 were excluded during the first title and abstract screening round.

Table 2 presents further information on the articles screened from each of the eight journals targeted in this review, as well as interrater-reliability values based on percent agreement and Cohen's kappa (two independent ratings on 5,048 reports). There was almost perfect agreement between the two raters' judgements on two journals, κ = 0.89–0.90, substantial agreement for four journals, κ = 0.72 and 0.79 and moderate agreement on the remaining two journals, κ = 0.44 and 0.58 (Landis and Koch, 1977). Percent agreement ranged from 68 to 98%.

**Table 2:** Summary of the procedure per journal (period: January 1998–September 2018).

**Journal**	**Number of articles screened**	**First title and abstract screening**				**Second abstracts screening^**b**^**	**Full text screening^**c**^**
		**Number of included articles^**a**^**	**Interrater-reliability**				
			**% agreement**	**Cohen's kappa**			
				**Estimate**	**95% CI**		
Canadian Journal of School Psychology	307	81	98	0.90	(0.94; 0.98)	15	2
International Journal of School and Educational Psychology	149	43	98	0.89	(0.95; 0.99)	14	1
Journal of Applied School Psychology	344	48	88	0.78	(0.63; 0.93)	6	2
Journal of School Psychology	685	32	88	0.79	(0.61; 0.96)	8	2
Psychology in the School	1,430	220	87	0.76	(0.69; 0.83)	20	7
School Psychology International	732	102	84	0.72	(0.60; 0.83)	13	4
School Psychology Quarterly	645	38	74	0.58	(0.38; 0.78)	9	3
School Psychology Review	756	59	68	0.44	(0.26; 0.62)	5	1
Total	5,048	623	–	–	–	90	22

Eligibility criteria:

a “school psychology” or “counseling” in the school context was explicitly mentioned in title and/or abstract. Editorials, commentaries, book and test reviews were excluded.

b “evaluation,” “effect,” “effectivity,” “efficacy,” and/or “effectiveness” were explicitly mentioned in the abstract.

c Articles provided explicit definitions and/or operationalizations of the effects.

*CI, confidence interval*.

In the second screening stage, further 533 articles were excluded as they did not meet the inclusion criteria for this review (see Figure 1). The remaining 90 articles moved on to the full text screening stage. In this final step of the selection process, we identified 22 articles with explicit definitions or mention of effects of school psychological services. The remaining 68 articles were excluded (see Figure 1).

### Percentage of Articles on the Effects of School Psychological Services

To answer research question 1, two rounds of title and abstract screening revealed that 1.78% of articles (i.e., 90 over a total of 5,048) published between January of 1998 and September of 2018 in the eight peer-reviewed journals targeted by this review explicitly referred to the measurement of effects of school psychological services in their title or abstract. However, only 0.43% (i.e., 22 over a total of 5,048) included definitions of effects of school psychological services or described outcomes used to evaluate school psychological services based on full text screening. See Table 2 for the number of included articles per journal.

### Type and Content of Articles on the Effects of School Psychological Services

Research question 2 intended to explore what type of articles had been published on the measurement of effects of school psychological services. Of the 22 articles that met inclusion criteria for this review, we identified 12 empirical research reports, six theoretical/conceptual articles, and four meta-analyses published between 1998 and 2015. The content covered varied considerably from article to article and is described with a short summary of each report in Tables 3A–C.

**Table 3A:** Summary of included empirical articles.

**References**	**Content**				**Effects of school psychological services**	
	**Study design**	**Participants**	**Country**	**Scope of investigation**	**Definition**	**Operationalization**
Chafouleas et al. (2002)	Survey	School psychologists (*N* = 189)	U.S.	Supervision and evaluation of school psychological work	Evaluation as a determination of the significance of an individual's professional skill (=implicit definition)	Non-standardized questionnaire on perception of and satisfaction with current evaluation practice (unspecified number of items)
Kikas (2003)	Survey	9th- and 12th graders (*N* = 433)	Estonia	Satisfaction with school psychological service, characteristics of students visiting school psychologists	Effect = perceived quality from students' perspective; no specific criterion mentioned	Non-standardized questionnaire assessing the quality of school psychological work (one item)
Proctor and Steadman (2003)	Survey	School psychologists (*N* = 63)	U.S.	Perceived effectiveness of counseling (=one aspect of the survey)	Effectiveness = school psychologists' self-disclosure on belief in making a significant difference at school/with students/with teachers	Non-standardized questionnaire on perceived effectiveness (six items)
Gilman and Gabriel (2004)	Survey	Teachers (*N* = 1,533) and administrators (*N* = 90)	U.S.	Satisfaction and helpfulness of school psychological services for students and educators	Effect = perceived quality from teachers' and administrators' perspective; no specific criterion mentioned	Non-standardized questionnaire assessing satisfaction with and helpfulness of school psychological work (two items)
Lepage et al. (2004)	pre-/post-test follow-up design without control-group	School psychology graduate students (*N* = 24)	U.S.	Evaluation of a 4-year consultation training program	Effect = behavioral change in clients (several behavioral problems were included), and preschool teachers' as well as parents' satisfaction with counseling	Non-standardized measures: repeated observations of client behavior rated by school psychologists, and questionnaire on clients' satisfaction and perceived quality (12 items)
Pérez-González et al. (2004)	Instrument development/Survey	Teachers (*N* = 157)	Spain	Construction and validation of an instrument to evaluate school psychologists' skills relevant for teacher consultation from teachers' perception	From teachers' perspective a school psychologist should offer expert knowledge concerning interventions, coordinate, initiate and follow-up interventions (= implicit definition)	–
Stobie et al. (2005)	Survey	School psychologists (*N* = 31)	U.K.	Use and evaluation of solution-focused practices	Effect = attainment of short- and long-term goals, satisfaction of the client with the outcome, and client-therapist relationship	Non-standardized questionnaire concerning the criteria used to evaluate effectiveness of counseling (nine items)
Hawken (2006)	pre-/post-test design without control-group	6th to 8th graders at-risk for poor peer relations and low academic achievement (*N* = 10)	U.S.	Evaluation of a school-wide prevention program after at least 6 weeks of intervention; program was implemented by teachers, administrators, and school psychologists	Effect = decrease of discipline referrals and increase in academic achievement; no data on achievement measures presented	Number of discipline referrals per week; formative standardized measurements (e.g., teacher interviews, self-disclosure) to modify intervention (=only recommended by the authors but no actual data reported)
Yeo and Choi (2011)	pre-/post-test follow-up control-group design	8- to 12-year-old students at-risk (*N* = 95)	Singapore	Evaluation of a 12-session cognitive-behavioral group therapy delivered by one school psychologist	Effects = increase of self-esteem, self-control, peer-relationship, social skills, and class-room behavior	Standardized problem-specific questionnaires of several data sources at three measurement occasions [rating scales on self-esteem, impulsive and self-controlled behavior, student report (in sum 70 items) and teacher report (in sum 33 items)]
Millar et al. (2013)	Survey	School psychologists (*N* = 34)	Canada	Feasibility examination of a professional development training for school psychologists to provide school-based intervention services for students with anxiety symptoms	Effects = capacity of school psychologists to provide mental health services; number of students that receive evidence-based intervention	Non-standardized survey on the perceived impact of the professional development practice on school psychologists own professional practice
Ogg et al. (2013)	Survey	School psychologists (*N* = 217)	U.S.	Evaluation of assessment practice with students with ADHD (=one aspect of the survey)	Effect = results of progress monitoring and development of outcome variables (both formative and summative), and assessment of intervention integrity	Non-standardized questionnaire on assessment practices with ADHD students with three items concerning outcome evaluation (three items); standardized measurements of adaptive functions and functional behavior analysis (=only recommended by the authors but no actual data reported)
Vu et al. (2013)	Randomized controlled study with control group	Teachers (*N* = 1,440)	U.S.	Evaluation of instruction consultation teams to support teachers with struggling students	Effect = teachers' perception of their own instructional practices, collaboration among school staff, own efficacy to affect student performance and job satisfaction	Four adapted or self-developed scales to measure teachers' perceptions of teaching practices (18 items), collaboration (10 items), self-efficacy (16 items) and job satisfaction (4 items).

*Definitions = descriptions used by the authors. If there was no explicit definition in the article, an implicit definition was concluded based of the procedure used*.

**Table 3B:** Summary of included theoretical or conceptual articles.

**References**	**Content**	**Effects of school psychological services**	
		**Definition**	**Operationalization**
Bradley-Johnson and Dean (2000)	Recommendation for school psychological services to shift from single-case work to preventive system-level work with teachers and the use of a systematic evaluation of services	Effect = evaluating teaching materials and procedures, intervention programs, and services (=implicit definition)	–
Durlak (2002)	Summary of the procedure used by a task force in the U.S. to evaluate the magnitude of the effects of school-related interventions	Effect of an intervention = changes in child adjustment (e.g., problems, symptoms, competences, grades) reaching important effect sizes	Recommendation to use standardized measurements of self-, parent-, and teacher ratings of symptoms and competencies as well as grades, achievement test scores, and clinical diagnostics
Strein et al. (2003)	Application of a public health model to school psychological service and research; proposed shift from single-case to system-level work	Recommendation to define effects as increase or decrease in the incidence and prevalence of outcomes at the school-level (e.g., grade retention, disciplinary referrals, performance on tests) instead of evaluation of effects with students with academic and behavioral problems	–
Hughes and Theodore (2009)	Conceptual framework how school psychologist can implement psychotherapy in school settings	Definition of school psychology as psychotherapeutic intervention to support academic and social development; effects = development of symptoms	Recommendation to use students' self-disclosure on symptom distress, behavioral observation of teachers, and standardized questionnaires
Nicholson et al. (2009)	Summary of possible negative effects of psychotherapy and counseling used by school psychologists based on reviews, intervention studies, and meta-analyses	Effects = development of symptoms	Disorder- and problem-specific outcomes (e.g., anxiety, depression, substance abuse no mention of standardized measures)
Morrison (2013)	Description of principles and methods in performance evaluation of school psychologists based on recommendations by the National Association for School Psychologists (NASP)	Effect = positive impact of school psychological services on student outcomes; recommendations for evaluating student outcomes and school psychologists' performance	Performance appraisal rubrics and rating scales to evaluate school psychologists adapted from instruments for teachers; repeated measures of students' outcomes using standardized items in single-case designs

*Definitions = descriptions used by the authors. If there was no explicit definition in the article, an implicit definition was concluded based of the procedure used*.

**Table 3C:** Summary of included meta-analysis.

**References**	**Content**			**Effects of school psychological services**	
	**Included studies**	**Years considered**	**Scope of investigation**	**Definition**	**Operationalization**
Prout and Prout (1998)	School-based psychological intervention studies with pre-/post-test-control-group designs (*N* = 17)	1985–1994	Direct counseling and psychotherapy group interventions; no limitations on the person carrying out the intervention; comparison of treatment type (cognitive-behavioral, relaxation, and skills)	Effect = development of symptoms related to problems to function in school	Disorder- and problem-specific outcomes (e.g., anxiety, depression, social skills) of several data sources (e.g., self-disclosure, grade or test scores, behavior observations no limitation on standardized measures)
Reddy et al. (2000)	Child and adolescent consultation studies (*N* = 35)	1986–1997	Comparison of behavioral, organizational developmental, and mental health consultation in school, home, and/or community for children from birth to 18 years of age; no limitations on design and the person carrying out the consultation	Effect = changes in behavior, mental health, or organizational communication, depending of type of consultation after receiving indirect service for parents, teachers, and other professionals	Problem-specific outcomes of clients, consultation, and systems no limitation on standardized measures
Reese et al. (2010)	School-based pre-/post-test-control-group designs reported in dissertations (*N* = 65)	1998–2008	School-based psychotherapy and counseling; thesis published electronically at a PhD-Server; no limitations on the persons carrying out the intervention; comparison of treatment type (cognitive-behavioral, relaxation, and skills)	Effect = development of symptoms after attending counseling understood as a psychotherapeutic intervention [analog to Prout and Prout (1998)]	Disorder- and problem-specific outcome measurements of several data sources (e.g., self-disclosure of students, reports of psychologists, teacher, parents no limitation on standardized measures)
Boudreau et al. (2015)	Studies measuring the effectiveness of peer/mediated pivotal response treatment to increase social-communication skills for children with autism spectrum disorders	1995–2008	Peer/mediated pivotal response treatment for school-aged children with an autism spectrum disorder diagnosis; single-subject design studies, although inclusion criteria not limited to this study-type	Effect = increases in the frequency of social-communication behavior of the target child with autism spectrum disorder	Direct behavioral observations using behavioral coding schemes, teacher questionnaires and pre-post language samples

Within the 12 *empirical articles*, eight articles used a survey technique (in one article the survey is part of an instrument development method) and four articles described longitudinal intervention studies. The participants of the survey were mostly school psychologists (5 of 8 articles), one survey was done with students and two with teachers. The four (quasi-)experimental longitudinal studies investigated the effects of interventions for students (2 of 4), school psychology students (1 of 4) and teachers (1 of 4). Based on different aspects of the effects of school psychological services investigated by each study, the content covered across articles can be summarized as follows: (1) articles about school psychological evaluation practices in general from school psychologists' perspective (4 of 12: Chafouleas et al., 2002; Stobie et al., 2005; Millar et al., 2013; Ogg et al., 2013), (2) one article on the evaluation of school psychological skills from teachers' perspective (Pérez-González et al., 2004), (3) articles on the efficacy and/or effectiveness of special interventions or trainings (3 of 12: Lepage et al., 2004; Hawken, 2006; Yeo and Choi, 2011), and (4) articles about satisfaction or perceived effectiveness of school psychological services (4 of 12: Kikas, 2003; Proctor and Steadman, 2003; Gilman and Gabriel, 2004; Vu et al., 2013).

Of the six *theoretical/conceptual articles*, three reports contained conceptual frameworks of the effects of school psychological services (Bradley-Johnson and Dean, 2000; Strein et al., 2003; Hughes and Theodore, 2009), two articles recommended methodological proposals to assess effects of services (Durlak, 2002; Morrison, 2013), and one critically discussed possible positive and negative effects of school psychological services (Nicholson et al., 2009).

Within the category *meta-analyses*, three reports summarized effect sizes of 17 (Prout and Prout, 1998) and 65 empirical group studies (Reese et al., 2010) and five single-subject-design studies (Boudreau et al., 2015) on direct school-based psychological services respectively and one investigated the overall effect of 35 studies on indirect services (Reddy et al., 2000). No limitation was placed on the persons carrying out the intervention, type of services, or the setting. Hence, there was no explicit focus on school psychology in any of the meta-analyses.

### Definitions of Effects of School Psychological Services

The definitions used to operationalize effects of school psychological services within the included 22 articles (see Table 3) indicate four thematic clusters (research question 3): (1) development of various psychological outcomes at the student level, (2) development of school/system-level outcomes, (3) consumer satisfaction, and (4) no explicit definition.

In relation to the first thematic cluster, most articles (i.e., 11 of 22) defined the effects of school psychological services as *development of psychological outcomes* at the student level. Three empirical articles (Lepage et al., 2004; Yeo and Choi, 2011; Ogg et al., 2013), four theoretical/conceptual articles (Durlak, 2002; Hughes and Theodore, 2009; Nicholson et al., 2009; Morrison, 2013), and all four meta-analyses (Prout and Prout, 1998; Reddy et al., 2000; Reese et al., 2010; Boudreau et al., 2015) fell into this category. However, given the fact that the four meta-analyses included counseling and consultation studies not necessarily delivered by school psychologists, the results should be interpreted with caution. Psychological outcomes addressed in the included articles predominantly focused on symptoms of mental health and/or academic or social competencies of students with disorders or at-risk students, depending on the context of the study. Mostly, development was equated to a positive change of these outcomes as evidenced through an increase or decrease of outcome scores based on data provided by pre-/post-test study designs. While some articles focused on development in a single subject or participant group receiving an intervention, others compared the development of an intervention group to a control group.

The second thematic cluster defined effects of school psychological services as the *development of school/system-level outcomes* and was represented by four of the 22 included articles (empirical: Hawken, 2006; Millar et al., 2013; Vu et al., 2013 theoretical/conceptual: Strein et al., 2003). These articles focused on the number of disciplinary referrals, grade retention frequency data, the capacity of school psychologists to provide services and the number of students receiving evidence-based interventions, and teachers' perceptions of the effects of consultation teams on their work and job satisfaction. A positive development indicating the effectiveness of school psychological services was therefore equated with a decrease or increase of these variables. The meta-analyses by Reddy et al. (2000), that was already categorized in the first cluster, can also be included within this category of school-level effects as it measures the overall effect of consultation on organizational development (e.g., improvement in communication or climate) in addition to other outcomes.

The third thematic cluster that defined effects as *consumer satisfaction* was solely represented by three empirical articles. While two studies focused on clients' perceived quality of the received service (Kikas, 2003; Gilman and Gabriel, 2004), the remaining study used school psychologists' self-disclosure of service efficacy as an outcome measure (Proctor and Steadman, 2003).

Finally, three of the 22 articles *did not give any explicit definition* of the effects of school psychological services (empirical: Chafouleas et al., 2002; Pérez-González et al., 2004; theoretical/conceptual: Bradley-Johnson and Dean, 2000). Implicitly, these articles referred to consumer satisfaction and evaluation practices as part of school psychological professional standards. Another article that we were unable to allocate into one of the categories was the study by Stobie et al. (2005). The authors defined effects of school psychological services in a broad manner as the attainment of goals without naming specific outcome variables.

### Operationalization of Effects of School Psychological Services

The instruments used to investigate effects (research question 4) were mainly disorder- and problem-specific measurements or satisfaction surveys. Questionnaires with rating scales were the most commonly used instruments. Common questions, for example, asked users about the helpfulness of and satisfaction with school psychological services for children and/or educators (e.g., Gilman and Gabriel, 2004) or the overall quality of the work of school psychologists (e.g., Kikas, 2003). Similarly, school psychologists' self-perception was measured by investigating, for example, whether practitioners believed that they were making a difference at their school, that they were effective with students and teachers, and whether they thought that teachers, parents, and administrators were knowledgeable about their skills and abilities (e.g., Proctor and Steadman, 2003). Ten of the 12 empirical articles used non-standardized questionnaires specifically developed or adapted for each study. The questionnaires used a wide range of number of items to evaluate effects, that is, one item in Kikas (2003) up to 18 items in Vu et al. (2013). Only one empirical paper used standardized questionnaires (Yeo and Choi, 2011), and one study used a behavioral observation technique (Lepage et al., 2004). All four meta-analyses included standardized and non-standardized outcome measures from several data sources (questionnaires, behavioral observation, school or clinical reports). The two theoretical/conceptual articles describing procedures and methods for evaluating school psychological services and school-related interventions recommended using standardized measurements (Durlak, 2002; Morrison, 2013).

## Discussion

The present study aimed to contribute to a better understanding of the measurement of effects of school psychological services by conducting a scoping review of scientific articles published between 1998 and 2018 in eight leading school psychology journals. Only 0.43% of all articles published within this period (i.e., 22 over a total of 5,048) met the inclusion criteria for our review of providing definitions and/or operationalizations of the effects of school psychological services. This shows that measurement of effects of school psychological services has, to date, been addressed in a very limited way by the scholarly community in the field of school psychology. This is surprising giving the fact that professional practice guidelines ask school psychologists to evaluate the efficacy of their services to ensure quality standards. However, addressing this issue is a complex task given the multiple users, intervention methods, and psychological needs involved in school psychological practice (e.g., paradox of school psychology, Gutkin and Conoley, 1990). This might explain why such a limited percentage of past research has tried to tackle this issue. Moreover, given the difficulties researchers might probably neglect to collect primary data on the effects of school psychology, opting instead to focus on aspects linked to school psychological work such as the effects of interventions or the usability of diagnostic tools. Consequently, if these studies do not use our keywords in their abstracts, they are not included in our study.

Nevertheless, the 22 studies that we identified provide a starting point to guide school psychologists in evaluating the effects of some of the dimensions of their work. The six theoretical/conceptual articles lay the groundwork by proposing frameworks, recommending methodological steps, and highlighting critical aspects that need to be considered when evaluating the effects of school psychological services. Three empirical studies conducted by researchers in experimental settings as well as four meta-analyses report information on measuring effects of school psychological services with the limitation that interventions were not necessarily carried out by school psychologists. In coherence with Andrews (1999), this evidence describes the impact school psychological services may have under ideal conditions in experimental settings. In fact, only nine of the 22 studies included in our review were delivered with school psychologists. This result highlights a research gap in the field of “effectiveness-studies” (Andrews, 1999), this is, the application of methods supported by experimental research findings in practical non-experimental real-world settings. If school psychologists are required to engage in evidence-based practices and evaluate the efficacy of their work (White and Kratochwill, 2005; Skalski et al., 2015), it is imperative that they can rely on scientific evidence obtained from real-world studies conducted with practicing school psychologists, not only on findings from experimental studies implemented by researchers. Empirical (quasi-) experimental studies conducted by researchers and/or practitioners with school-aged samples were the most frequently published papers in the eight leading school psychology journals from 2000 onward (Villarreal et al., 2017; cf. Begeny et al., 2018). Further research should aim to confirm the effects of these (quasi-)experimental studies in non-experimental settings by including practicing school psychologists as potential professionals delivering the investigated interventions. The contributions by Forman et al. (2013) with respect to the development of implementation science in school psychology can provide a valuable starting point in this respect. Also, the ideas proposed by Nastasi (2000, 2004) of creating researcher-practitioner partnerships are important to move the field forward and enable school psychologists to function as scientist-practitioners (Huber, 2007). Only if researchers and practitioners work together to increase the adoption of evidence-based practices, common obstacles, such as the reduced importance of research in school psychological practice, can be tackled to improve service delivery (Mendes et al., 2014, 2017).

In terms of the definitions used to refer to effects of school psychological services, most articles focused on the development of psychological outcomes at the student level such as symptoms, behaviors, or competences (i.e., 11 of 22). Only a few studies understood effects as the development of school/system-level outcomes such as discipline referrals (i.e., 4 of 22), consumer satisfaction of service users (i.e., 3 of 22) or provided no explicit definitions (i.e., 4 of 22). No studies with an economic perspective on the definition of effects were identified. Thus, available definitions capture single aspects of the multiple dimensions involved in school psychological practice. The effects of direct student-level services are undoubtably a core component of the efficacy of school psychological services, but more research is needed to guide practitioners with respect to the evaluation of indirect services at the school level and indirect mediation effects of adults, who work with school psychologists, on outcomes at the student level (cf. Gutkin and Curtis, 2008). Following the suggestions by Strein et al. (2003) of adopting a public health perspective may aid researchers in adopting a broader focus of the evaluation of effects of school psychological services.

Regarding the operationalization of effects of school psychological services, we identified a mismatch between theoretical and conceptual proposals of what “should be done” and “what has actually been done” as reported by the available evidence. While theoretical/conceptual articles recommended the use of standardized measures, in practice ten of 12 empirical studies used non-standardized instruments, only in some cases offered information on the reliability of these instruments and, on occasions, only included one item. Also, the four meta-analyses did not limit their inclusion criteria to studies using standardized measures. This finding is concerning, as the methodological quality of instruments determines the extent to which effects of school psychological services may be captured. Thus, empirical research in this field should lead by example and use reliable and valid assessment instruments.

Our results need to be interpreted with caution taking at least two limitations into account. First, to be included in our scoping review, studies had to explicitly refer to the concepts “school psychology” or “counseling” in the school context in their title and/or abstract. We were interested in exploring how the effects of school psychological services are conceptualized and measured within this applied field of psychological research. However, school psychology often relies on contributions from other related research fields (e.g., counseling research, clinical psychology). Given our eligibility criteria this kind of research was not included in our review, although it also plays a role in guiding evaluations of the effects of school psychological services. Readers should, therefore, keep in mind that the evidence summarized in this review only refers to research explicitly associated with the field of school psychology. This limitation is especially important to keep in mind for international readers, as different professional titles are used worldwide to refer to psychologists that cater for needs of students, teachers and other school staff. In European countries, for example, the term “educational psychologist” is often used as a synonym of “school psychologist.” As our review focused on articles that explicitly mentioned the terms “school psychology” or “counseling” in the school context in their title and/or abstract, it is possible that we excluded relevant publications using derivations of the term “educational psychology.”

Second, our review is limited to past research published in eight leading school psychology journals that predominantly focus on contributions by researchers and samples located in the U.S (see Begeny et al., 2018 for a similar approach). This decision certainly limits the generalizability of our results. Therefore, we alert the reader to keep in mind that our findings might be showing an incomplete picture and encourage future research to replicate our study with additional information sources.

## Conclusion

This scoping review contributes toward a better understanding of how the measurement of effects of school psychological services has been conceptualized by past research in major school psychology journals. The results show very limited research on this topic despite the relevance of evaluation in guidelines of school psychological practices. We systematically identified, summarized, and critically discussed the information from 22 articles that may serve as a guide for policymakers and school psychologists aiming at evaluating the effects of their services informed by scientific evidence. According to our results, the definitions of the effects of school psychological services only capture some aspects of the multiple dimensions involved in school psychological practice and the methodological quality of the instruments used to assess the efficacy needs to be improved in future studies. It seems that school psychologists can rely on a large amount of interventions with experimental research support, but studies investigating fidelity and effects of these interventions in practical non-experimental school psychological settings are less common. Overall, the results represent a starting point to conceptualize measurement of effects of school psychological services. More research is urgently needed to provide school psychologists with evidence-based tools and procedures to assess the effects of their work and ensure the quality of school psychological services.

## Author Contributions

The initial identification process on the journals' homepages and the first screening of titles and abstracts was done by BM. The second screening of the abstracts, the full text screening, and data charting was done by BM. Syntheses of our results were done by BM, AH, NV, and GB. All authors contributed to the article and approved the submitted version.

## Conflict of Interest

The authors declare that the research was conducted in the absence of any commercial or financial relationships that could be construed as a potential conflict of interest.

## Footnotes

^1^Reports published in the *Canadian Journal of School Psychology* and the *International Journal of School and Educational Psychology* were screened in January 2021 during the review process as recommended by one of the reviewers to extend our search.
